# Anti-CD7 allogeneic WU-CART-007 in patients with relapsed/refractory T-cell acute lymphoblastic leukemia/lymphoma: a phase 1/2 trial

**DOI:** 10.21203/rs.3.rs-4676375/v1

**Published:** 2024-08-05

**Authors:** Armin Ghobadi, Ibrahim Aldoss, Shannon Maude, Deepa Bhojwani, Alan Wayne, Ashish Bajel, Bhagirathbhai Dholaria, Rawan Faramand, Ryan Mattison, Anita Rijneveld, C. Zwaan, Frisco Calkoen, Andre Baruchel, Nicolas BOISSEL, Michael Rettig, Brent Wood, Kenneth Jacobs, Stephanie Christ, Haley Irons, Ben Capoccia, Justo Gonzalez, Tony Wu, Maria del Rosario, Alexander Hamil, Ouiam Bakkacha, John Muth, Brett Ramsey, Eileen McNulty, Matthew Cooper, Jan Baughman, Jan Davidson-Moncada, John DiPersio

**Affiliations:** Washington University School of Medicine; City of Hope National Medical Center; University of Pennsylvania; CHLA; Children’s Hospital Los Angeles; Peter MacCallum Cancer Center; Vanderbilt University Medical Center; Moffitt Cancer Center; University of Wisconsin Carbone Cancer Center; Erasmus Medical Center; Erasmus Medical Center-Sophia Children’s Hospital; Prinses Máxima Centrum; University Hospital Robert Debré and University Paris Diderot; aphp hôpital saint louis paris; Washington University in St Louis; University of Southern California; Mersana Therapeutics; Washington University School of Medicine; Wugen; Wugen; Wugen; Wugen; Wugen; Wugen; Wugen; Wugen; Wugen; Wugen; Wugen; Alviso Clinical Research; Wugen; Washington University in St. Louis

## Abstract

Relapsed/refractory T-cell acute lymphoblastic leukemia (ALL)/lymphoma (LBL) represent a significant unmet medical need. WU-CART-007 is a CD7-targeting, allogeneic, fratricide-resistant chimeric antigen receptor T cell product generated from healthy donor T cells. WU-CART-007 was evaluated in a phase 1/2 study with a 3 + 3 dose-escalation design followed by cohort expansion in relapsed/refractory T-ALL/LBL. Patients received one infusion of WU-CART-007 after standard or enhanced lymphodepleting chemotherapy. The primary objectives, to characterize safety and assess the composite complete remission rate, were met. Of 26 patients enrolled, 13 received the recommended phase 2 dose (RP2D) of 900 million cells of WU-CART-007 with enhanced lymphodepletion. The most common treatment-related adverse event was cytokine release syndrome (88.5%; 19.2% grade 3–4). Biochemical abnormalities consistent with grade 2 hemophagocytic lymphohistiocytosis were seen in one patient (3.8%). Grade 1 immune effector cell-associated neurotoxicity syndrome events (7.7%) and one grade 2 acute graft-vs-host disease event occurred. Grade 5 events (11.5%) were due to fungal infection and multi-organ failure. The composite complete remission rate was 81.8% among 11/13 patients evaluable for response at the RP2D. WU-CART-007 at the RP2D demonstrated a high response rate in patients with relapsed/refractory T-ALL/LBL and has the potential to provide a new treatment option. ClinicalTrials.gov registration: NCT04984356.

## Introduction

Relapsed/refractory T-cell acute lymphoblastic leukemia (T-ALL) and lymphoblastic lymphoma (T-LBL) carry a dismal prognosis with limited effective therapeutic options. Current standard chemotherapy regimens lead to poor outcome with 6 months median overall survival in responders (1).

Lymphoblastic lymphoma is the second most common type of non-Hodgkin’s lymphoma; 70–80% is of T-lymphoblastic origin, accounting for 25–35% of childhood and adolescence cases (2). Like T-ALL, LBL is characterized by expression of terminal deoxynucleotidyl transferase, CD2, cytoplasmic and/or surface CD3, CD5, and, importantly in this context, expression of CD7 (3, 2).

The World Health Organization (WHO) classifies T-ALL and T-LBL as the same disease (4, 5, 6, 7, 8) and the clinical approach to treating them is similar. There is no standard of care treatment for adult patients with relapsed disease; thus, there is an unmet medical need, with median survival of only 6 months and < 7% of patients surviving at 5 years (1, 9).

Only one drug has been approved for relapsed/refractory T-ALL/LBL since 2005. Nelarabine was approved based on complete response/complete response with incomplete hematologic recovery rates of 23% in 39 pediatric patients and 21% in 28 adults. Neurologic toxicity was dose-limiting for both populations (10).

Cluster of differentiation (CD) 7 is the most frequently expressed T-cell antigen, expressed in > 95% of newly diagnosed patients with T-ALL/LBL (3). CD7 is consistently expressed at diagnosis, relapse, and minimal residual disease in adult and pediatric cases (11).

Despite success in B-cell malignancies, development of autologous chimeric antigen receptor (CAR)-T cell therapy for T-cell cancers is complicated by high risk of malignant cell contamination of the drug product in the autologous setting, which allogeneic sources obviates, and induction of fratricide. An effective “off-the-shelf” therapy has the potential to avoid or reduce the number of early patient deaths from rapidly progressive disease and toxicities of bridging therapies that occurs during the time required to produce autologous CAR-T cells (12).

WU-CART-007 is an allogeneic CD7-directed, genetically modified, fratricide-resistant CAR-T cell product under development for treatment of CD7-positive hematologic malignancies. To manufacture WU-CART-007 drug product, T cells generated from healthy donors are CRISPR-Cas9 edited to delete CD7 and T-cell receptor alpha constant gene (TRAC), transduced with a second-generation CAR targeting CD7, expanded, purified to remove residual T-cell receptor α/β-positive cells, and cryopreserved (Extended Data Fig. 1; 13). Proof-of-concept of this approach, allogeneic-sourced CD7 targeting CAR-T cells, has been previously reported in small single site studies (14, 15).

We report results from the primary analysis of a global multi-center phase 1/2 dose escalation and expansion study to assess the safety and efficacy of WU-CART-007 in relapsed/refractory T-ALL/LBL.

## Results

**Figure 1:**
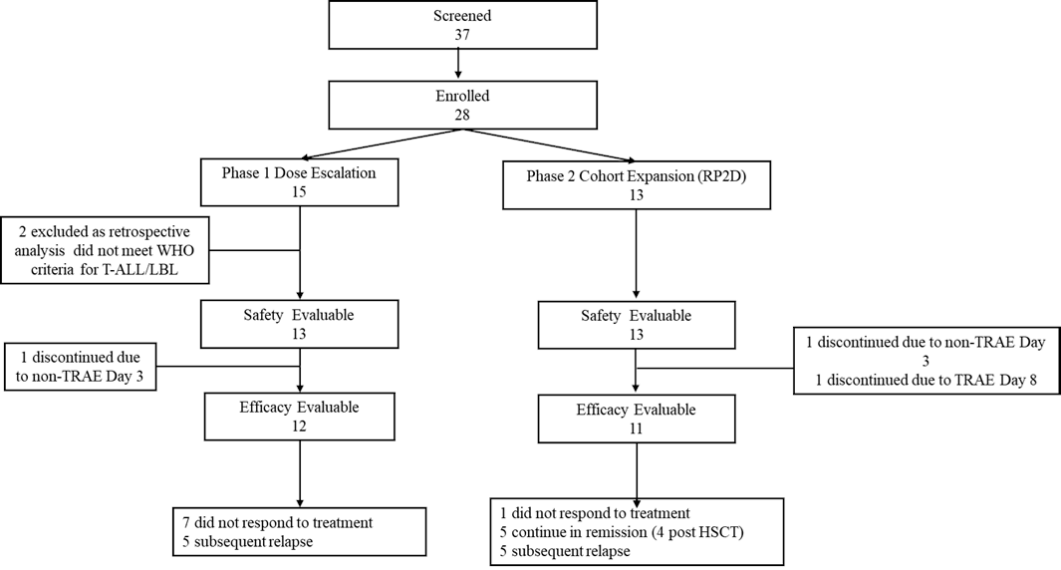
Patient Disposition: CONSORT Diagram. RP2D, recommended Phase 2 dose; WHO, World Health Organization; TRAE, treatment-related adverse event; HSCT, hematopoietic stem cell transplantation.

Thirty-seven patients were screened, of which 28 were enrolled (February 2022 to December 2023): 15 in dose escalation, and 13 in cohort expansion at the RP2D of 900 million (M) WU-CART-007 cells following enhanced lymphodepletion (Fig. 1). Two patients in dose level 3 were excluded from analyses based on not meeting diagnostic criteria for T-ALL/LBL as defined by WHO (safety for these two patients is presented in Extended Data Table 4). Three patients were excluded from efficacy analyses (1 in dose level 3 and 2 in phase 2) as they were not response-evaluable (no post-baseline disease assessment; discontinued day 3 (n = 2) and day 8 (n = 1)). These patients are included in the safety evaluable population.

### Patient characteristics

The median age was 30 years (range, 14–69) (Table 1). Five adolescents were enrolled, all treated at the RP2D. Patients had a median of 4 prior lines of therapy (range 2–7), with 26.9% (7/26) primary induction failure; 38.5% patients (10/26) had undergone previous allogeneic hematopoietic stem cell transplantation, and 77% (20/26) had prior nelarabine therapy. Baseline disease burden consisted of extramedullary disease only in seven (26.9%) patients, and median bone marrow blast count was 50% (range, 5–95%) in patients with bone marrow disease.

### Safety

One dose limiting toxicity (dose level 3) of grade 5 encephalopathy associated with intracranial infection, deemed not related to WU-CART-007, occurred. After assessment of overall safety, the recommended phase 2 dose RP2D was defined as 900 million WU-CART-007 cells following enhanced lymphodepletion.

Twenty-five of 26 (96.2%) patients had ≥ one treatment-related adverse event, of whom 16 (61.5%) had ≥ grade 3 events (Table 3). The most common events were cytokine release syndrome (CRS; 88.5%), international normalized ratio increased (34.6%), blood fibrinogen decreased (30.8%), anemia (26.9%), blood bilirubin increased (23.1%); and pyrexia (23.1%).

The most common treatment-emergent serious adverse events were CRS (26.9%) and sepsis (11.5%) (Extended Data Table 2).

Grade 5 events occurred in 3 patients (11.5%) and included encephalopathy (600M) and sepsis (RP2D) secondary to invasive fungal infection, both not attributed to WU-CART-007 and occurred in patients who had an ANC of 0.0 prior to receiving WU-CART-007 infusion and/or received multiple doses of immunosuppressive agents as treatment for CRS/hemophagocytic lymphohistiocytosis (HLH; 16). Additionally, one grade 5 multi-organ dysfunction syndrome was reported as related to WU-CART-007, which occurred in the setting of rapid progression of the underlying highly-refractory disease of T-ALL.

Cytokine release syndrome was grade 1–2 in 69.2% patients, with grade 3 in three (11.5%) and grade 4 in two (7.7%). Grade 4 CRS completely resolved within 7 and 13 days, respectively. The median time to onset of CRS was 24 hours, with a median duration of three days, necessitating antipyretics, tocilizumab, and/or corticosteroids for management.

Hemophagocytic lymphohistiocytosis/macrophage activation syndrome event occurred in one patient (3.8%), was of grade 2 severity, and resolved with anakinra and steroids. This event occurred on day 8 and was characterized by transient laboratory abnormalities with no clinical sequelae.

Immune effector cell-associated neurotoxicity syndrome events occurred in two patients (7.7%) and were both grade 1.

Acute skin graft-vs-host disease (biopsy-confirmed) occurred in one patient who had no history of stem cell transplant, was grade 2, occurred two months after WU-CART-007 infusion at the recommended phase 2 dose, and was manageable with steroids to full resolution within 48 hours.

Five patients (19.2%) experienced grade 3 and 4 infection events irrespective of attribution at the recommended phase 2 dose; most cases were effectively managed using antimicrobial therapy.

### Efficacy

**Figure 2:**
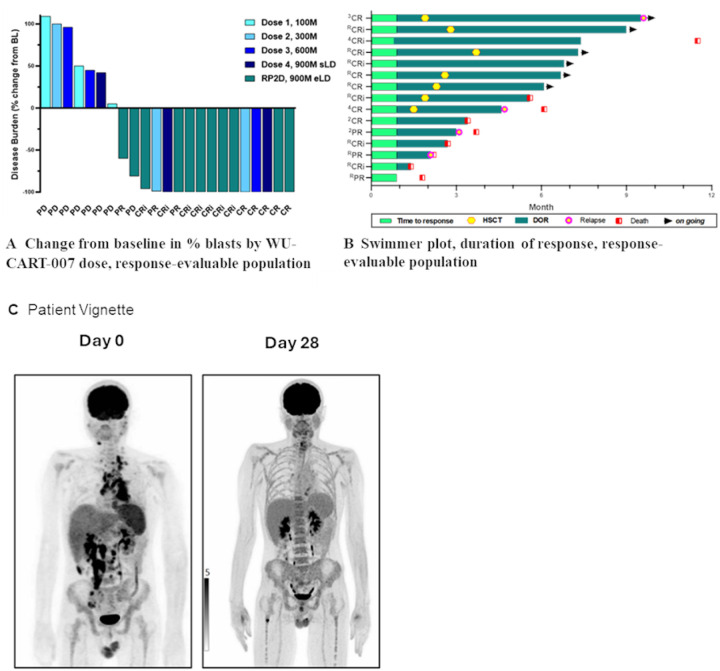
Best Overall Response by WU-CART-007 Dose and Duration of Response, Response-evaluable Population (A) Waterfall plot of best overall response by WU-CART-007 dose (B) Swimmer plot of duration of response. (C) 36-year-old male with a history of refractory T-LBL failed 2 lines of previous therapy enrolled on Phase 2 part of the study and treated at the recommended phase 2 dose. Patient achieved a complete and durable response. BOR, best overall response: CR, complete remission: CRi, complete remission with incomplete hematologic recovery; DOR, duration of response; HSCT, hematopoietic stem cell transplantation; PR, partial response (extramedullary disease only); PD, progressive disease; RP2D, recommended phase 2 dose

WU-CART-007 showed increased anti-tumor activity at each dose level after dose level 1, where there were no responses. Figure 2B presents a swimmer plot of percent change in blasts from baseline. Across all dose levels with 23 response-evaluable patients, the composite complete remission (CRc) rate was 52.2% and the objective response rate (ORR) was 65.2%.

At the RP2D, eleven of 13 patients were response-evaluable; among these, the CRc rate was 72.7% (8/11), and the ORR was 90.9% (Table 2). Median duration of response was not reached (95% confidence interval 0.5 – not estimable (NE), range 0.5–8.1 months) with median duration of follow-up not reached (95% confidence interval 1.4 – NE, range 1.4–9 months).

Ten patients received WU-CART-007 in the post-hematopoietic stem cell transplant relapse setting, with an ORR of 88.9% (8/9; 1 not evaluable) and an ORR of 100% (5/5) at the RP2D. Seven patients transitioned to hematopoietic stem cell transplant after WU-CART-007 treatment, all in complete remission; 80% (4/5) with T-ALL had undetectable minimal residual disease (MRD), including four treated at the RP2D. There were no engraftment delays reported in these patients; neutrophil recovery occurred at a median of 19 (range 13–25) days. Figure 2C demonstrates a representative patient treated at the RP2D achieving complete remission.

At the RP2D, response rates were comparable for patients with T-ALL and T-LBL, with a CRc in 87.5% (7/8) and 33.3% (1/3) and an ORR of 7/8 (87.5%) and 3/3 (100%), respectively.

Of patients with CRc, 83.3% (10/12) have MRD data available; 70% (7/10) were MRD undetectable and 30% (3/10) are MRD detectable. Median duration of response is not reached to date (95% CI: 1.8, NE) and 3.76 months (95% CI: 0.50, NE) for MRD undetectable and MRD detectable patients, respectively.

Five patients with extramedullary disease (EMD) were treated at the RP2D, three of whom had EMD only and two of whom had both EMD and bone marrow disease. These patients were heavily pre-treated (median prior lines of therapy 2, range 2–3) and 1 (20%) had received prior hematopoietic stem cell transplantation. The ORR in these patients was 80% (4/5; 2 CR/2 partial response [PR]). For patients that achieved PR, the decrease in disease burden was 78.6 and 87.6% by Lugano Classification.

### Cellular PK and Immune Cell Phenotyping

Expansion of WU-CART-007 cells at the RP2D peaked on day 10 in peripheral blood (median expansion 242,424 CAR copies/μg genomic DNA) and persisted out to day 90 (Fig. 2A). For comparison, expansion was determined in patients that received a dose of 900 million WU-CART-007 cells following standard lymphodepleting regiment (n = 3). Expansion was approximately seven-fold higher in the group receiving enhanced lymphodepletion (242,424 *vs*. 34,545 copies/μg genomic DNA) with a longer persistence (day 90 *vs*. day 28) (area-under-curve comparison in Extended Data Table 5). Of 20 patients sampled, none developed novel anti-human leukocyte antigen antibodies against the donor or anti-CAR specific antibodies.

Cytopenia, with gradual resolution, was observed in patients receiving enhanced lymphodepletion and 900 million WU-CART-007 cells (Extended Data Fig. 2A). Mean white blood cell counts reached 2,223 cells per μL and neutrophil counts reached 1,480 cells per μL by day 90. Circulating lymphocytes recovered more slowly, reaching 366 cells per μL mean by day 90. The percentage of normal T cells expressing CD7 declined after lymphodepletion and WU-CART-007 dosing, reaching a nadir concurrent with WU-CART-007 peak expansion on day 10 (Extended Data Fig. 2B). A population of CD7^neg^ normal T cells arose as the CD7^pos^ normal T cells declined, reaching 56 cells per μL on day 10.

A reduction in CD7^pos^ T-cell acute lymphoblastic leukemia and increase in WU-CART-007 with expression of cytotoxic markers was observed in peripheral blood, peaking at day 10 (Fig. 2, Extended Data Figs. 4 and 5). Circulating WU-CART-007 cells transitioned to a highly activated CD8^pos^ effector memory CD45RA^pos^ phenotype with low expression of exhaustion markers (Fig. 2, Extended Data Fig. 3).

## Discussion

For adults and children with T-ALL/LBL who relapse after initial therapy, salvage chemotherapy induces remission in only 20–40% of cases. No treatment besides nelarabine has been approved in this setting since 2005, with unsatisfactory response rates (10, 17). There is limited experience with CD7-directed CAR-T therapies in the T-ALL/LBL setting due to widespread antigen overlap in healthy T cells and the risk of CART-T cell fratricide. Preliminary results from a study of a CD7-targeted CAR-T, GC027, showed responses in 9/11 patients and manageable toxicity (15). Base-editing of healthy donor T cells transduced to express a CAR with specificity for CD7 is being evaluated in a phase 1 pediatric trial of T-ALL, with preliminary results in three children showing molecular remission and subsequent transplant in two, and fatal fungal complications in one patient (14). A strategy of sequential CD7 CAR-T therapy and haploidentical hematopoietic stem cell transplantation in 10 patients with relapsed/refractory CD7-positive leukemia or lymphoma yielded encouraging efficacy (18).

WU-CART-007 was generally well tolerated, with CRS, primarily grade 1–2, as the most frequently-occurring treatment-related adverse event. Grade 2 hemophagocytic lymphohistiocytosis event primarily consisted of biochemical abnormalities. Immune effector cell-associated neurotoxicity syndrome events were grade 1, and only one treatable grade 2 acute graft-vs-host disease was reported. Infections were more frequently observed with enhanced lymphodepletion, with most cases effectively managed using antimicrobial therapy. Two patients died due to invasive fungal infection early in treatment in the setting of persistent disease and after substantial treatment with immunosuppressive agents to manage immune-related toxicities. Infection surveillance and prophylactic antimicrobial therapy is essential in this heavily pre-treated population. One patient died in the setting of progressive disease; hence, patients with rapidly-progressing disease will be excluded from future enrollment as a mitigation strategy.

WU-CART-007 expansion was robust with persistence *in vivo*. Increasing intensity of lymphodepleting chemotherapy led to substantial increase in expansion and persistence, which were associated with higher objective response rate; at the 900 million cell dose, objective response rate was 66% (2/3) vs. 91% (10/11) for standard vs. enhanced lymphodepletion, respectively; however, the study is under powered for definitive evaluation.

No patients developed anti-human leukocyte antigen antibodies against the donor or anti-drug antibodies against the CAR construct.

For patients that relapse following a hematopoietic transplant, achieving a subsequent remission is even more challenging. Ten patients received WU-CART-007 in the post-hematopoietic stem cell transplant relapse setting, with a CRc rate of 88.9% (8/9), and an ORR of 100% (5/5) at the RP2D. Three of these patients received a subsequent hematopoietic stem cell transplant. In total, seven of 11 response-evaluable patients transitioned to hematopoietic cell transplantation after WU-CART-007, suggesting its potential to offer a successful bridge to transplant.

The results from this study demonstrate that the RP2D of WU-CART-007 provides clinically meaningful responses in patients with T-ALL/LBL, with a CRc rate of 72.7% and an ORR of 90.9%. Response rates were similar for patients with T-ALL and T-LBL. Based on the high response rate (as defined in the Simon 2 stage) seen at the RP2D, enrollment was completed in preparation of a registration-enabling study.

In conclusion, WU-CART-007 has demonstrated a high rate of response in heavily pre-treated patients with relapsed/refractory T-ALL/LBL, including in the post-hematopoietic stem cell transplantation setting; however, interpretation of these study results is limited by the small sample size and lack of comparator arm. This program has the potential to advance an off-the-shelf allogeneic CAR-T cell therapy and provide a new treatment option for relapsed CD7-positive T-cell malignancies such as T-ALL/LBL, which represent a significant unmet medical need.

## Online Methods

### Study Design

The study was approved by the Institutional Review Board at each institution and conducted in accordance with Good Clinical Practice, the Declaration of Helsinki, and local ethical and legal requirements. All patients/legal guardians provided written informed consent before enrollment. This study was registered at ClinicalTrials.gov: NCT04984356.

The following independent ethics committee or IRB provided approval of this study: Royal Melbourne Hospital Human Research Ethics Committee, Parkville, Australia; Comité de Protection des Personnes, Nord Ouest IV, Nantes, France; Central Committee on Research Involving Human Subjects, The Hague, Netherlands; Advarra IRB, Cincinnati, OH, USA; Children’s Hospital of Philadelphia IRB, Philadelphia, PA, USA; Vanderbilt IRB, Nashville, TN, USA; WCG IRB, Puyallup, WA, USA.

This was a first-in-human, open-label, phase 1/2 study. Phase 1 was a 3 + 3 dose-escalation design evaluating 4 dose levels of 100, 300, 600, and 900 million WU-CART-007 cells in cohorts of up to 6 patients to determine the maximum tolerated/maximum administered dose and the RP2D (Extended Data Table 1). The dose limiting toxicity evaluation period was 28 days following WU-CART-007 infusion. Patients received a preparative standard lymphodepletion conditioning chemotherapy regimen consisting of fludarabine 30 mg/m^2^/day and cyclophosphamide 500 mg/m^2^/day on days − 5, −4, and − 3 prior to WU-CART-007 infusion. Once the maximum administered dose was determined, 3 patients were enrolled to evaluate an enhanced lymphodepletion regimen, consisting of an additional dose of fludarabine 30 mg/m^2^/day (Day − 6) and increase in cyclophosphamide dose from 500 to 1000 mg/m^2^, to evaluate its effect on the pharmacokinetics of CAR-T cells.

During Phase 2, up to 20 patients with R/R T-ALL/LBL were to be enrolled to receive the same regimen of WU-CART-007 as per the recommended phase 2 dose (RP2D).

### Patients

#### Inclusion Criteria.

Patients were eligible if they met all the following criteria: (1) Evidence of relapsed or refractory T-ALL or T-LBL, as defined by World Health Organization (WHO) classification with bone marrow with ≥ 5% lymphoblasts by morphologic assessment or evidence of extramedullary disease at screening; (2) relapsed or refractory disease defined as at least one of the following criteria: a. Primary refractory: failure to achieve CR after induction chemotherapy, per investigator. b. Early Relapse: relapsed disease within 12 months of initial diagnosis. c. Late Relapse (relapsed refractory disease): relapsed disease after 12 months of initial diagnosis AND failure of re-induction therapy after disease recurrence. d. Relapsed or refractory disease after allogeneic transplant, and met the following criteria: i. There must have been histological confirmation of relapse after HSCT of T-ALL or T-LBL, ii. Undergone allogeneic HSCT > 90 days prior to enrollment from a match related or unrelated donor, cord blood donor, haploidentical, or autologous stem cells. iii. Off all immunosuppressive medications for a minimum of 2 weeks with the exception of physiologic doses of corticosteroids, iv. No prior history of grade 2 or greater (per Cairo-Bishop) veno-occlusive disease/sinusoidal obstruction syndrome (VOD), or active GvHD (see Exclusion Criteria 8 for exceptions); (3) adequate organ function, defined as: a. Hepatic and renal function: i. Hepatic transaminase (both alanine aminotransferase and aspartate aminotransferase levels ≤ 5 times the institutional upper limit of normal (ULN), ii. Total bilirubin level ≤ 1.5 times the ULN (unless the patient has a history of Gilbert’s Syndrome, in which case, total bilirubin must be ≤ 2.5 times the ULN), iii. Serum creatinine level ≤ 1.5 times the ULN or a calculated or measured creatinine clearance of ≥ 50 ml/min. b. Respiratory function: Must have had a minimum level of pulmonary reserve defined as pulse oxygenation > 91% on room air. c. Cardiovascular function: left ventricular ejection fraction ≥ 45% confirmed by echocardiogram or MUGA within 28 days of screening; (4) life expectancy > 12 weeks; (5) age: lower age limit of 12 years. Adolescents ages 12–17 were eligible for enrollment beginning at Dose Level 3 of the Dose Escalation phase, after review of safety, efficacy and pharmacokinetic data and after consultation the appropriate regulatory agencies; (6) Eastern Cooperative Oncology Group (ECOG)/Karnofsky performance status 0 or 1 at screening (adults age > 16) or Lansky Performance Status 60 and above (adolescents age ≤ 16); (7) ability to understand the nature of this study, comply with protocol requirements, and give written informed consent. For minors, legal guardian willingness to give written informed consent with patient assent, where appropriate; (8) willing to participate in study WUC-007-02 for long-term follow up; (9) patients of reproductive potential: All female study participants of reproductive potential must have had a negative serum or urine pregnancy test performed prior to start of lymphodepletion therapy. Study participants were also required to use adequate contraception during the study treatment period and for 12 months after the last dose of study drug; (10) the first 5 patients on study were to be screened and matched for potential allogeneic marrow transplant in the event of profound and persistent T-cell aplasia.

#### Exclusion Criteria.

Patients were excluded from study entry if any of the following criteria were met: (1) patients with concomitant genetic syndrome, such as patients with Fanconi anemia, Kostmann syndrome, Shwachman syndrome, or any other known bone marrow failure syndrome. Patients with Down syndrome were not excluded; (2) treatment with any prior anti-CD7 therapy; (3) unresolved toxicities from prior anticancer therapy, defined as having not resolved to baseline or to common terminology criteria for adverse events (AEs) (CTCAE) grade ≤ 1, with the exception of nausea or alopecia, or to the levels dictated in the inclusion/exclusion criteria; (4) patient had previously participated in any investigational research study and was being screened for participation within a period of 5 half-lives of the last dose of the investigational therapy; (5) active or latent hepatitis B or active hepatitis C, or any uncontrolled infection at screening; (6) human immunodeficiency virus-positive test within 8 weeks of screening; (7) serious active infection at the time of treatment, or another serious underlying medical condition that would impair the ability of the patient to receive protocol treatment; (8) presence of grade 2 to 4 acute or extensive chronic GvHD requiring systemic immunosuppression (steroids). Grade 1 GvHD not requiring immunosuppression and grade 2 skin GvHD if treated with topical therapy only was acceptable; (9) presence of other active cancers, or history of treatment for invasive cancer ≤ 3 years. Patients with Stage I cancer who had received definitive local treatment and were considered unlikely to recur were eligible. All patients with previously treated in situ carcinoma (i.e., noninvasive) were eligible, as were patients with history of nonmelanoma skin cancer; (10) patients with active central nervous system (CNS) leukemia involvement defined as CNS-3 by cerebrospinal fluid findings were not eligible unless CNS disease was cleared by negative cytology in 2 consecutive samples at least 1 week apart; (11) psychological, familial, sociological, or geographical conditions that did not permit compliance with the protocol; (12) pregnant or nursing (lactating) women; (13) the following medications were excluded: a. Steroids: Therapeutic systemic doses of steroids must have been stopped > 72 hours prior to WU-CART-007 infusion. However, the following physiological replacement doses of steroids were allowed: <12 mg/m2/day hydrocortisone or equivalent. b. Allogeneic cellular therapy: Any donor lymphocyte infusions must have been completed > 6 weeks prior to WU-CART-007 infusion. c. GvHD therapies: Any systemic drug used for GvHD must have been stopped > 4 weeks prior to WU-CART-007 infusion to confirm that GvHD recurrence was not observed (e.g., calcineurin inhibitors, methotrexate or other chemotherapy drugs, mycophenolate, rapamycin, thalidomide, or immunosuppressive antibodies such as anti-CD20 (rituximab), antitumor necrosis factor (anti-TNF), anti-interleukin 6 (anti-IL6) or anti-interleukin 6 receptor (anti-IL6R), Janus kinase (JAK) inhibitors, systemic steroids). d. Chemotherapy: i. Hydroxyurea must have been stopped > 24 hours prior to lymphodepletion. ii. The following drugs must have been stopped > 1 week prior to WU-CART-007 infusion and should not have been administered concomitantly or following lymphodepleting chemotherapy: vincristine, 6–mercaptopurine, 6-thioguanine, methotrexate < 25 mg/m2, cytosine arabinoside < 100 mg/m2/day, asparaginase (non-pegylated), and nelarabine. iii. The following drugs must have been within 5 half-lives of the last dose prior to WU-CART-007 infusion and all related toxicities resolved to grade 1 or baseline: salvage chemotherapy (e.g., clofarabine, cytosine arabinoside > 100 mg/m2, anthracyclines, cyclophosphamide, methotrexate ≥ 25 mg/m2), excluding the required lymphodepleting chemotherapy drugs. iv. Pegylated-asparaginase must have been stopped > 4 weeks prior to WU-CART-007 infusion. v. CNS prophylaxis treatment must have been stopped > 1 week prior to WU-CART-007 infusion (e.g., intrathecal methotrexate). e. Radiotherapy: i. Non-CNS site of radiation must have been completed > 2 weeks prior to WU-CART-007 infusion. ii. CNS-directed radiation must have been completed > 8 weeks prior to WU-CART-007 infusion. f. Anti T-cell monoclonal antibodies: Administration of any T-cell lytic or toxic antibody within 8 weeks prior to WU-CART-007 was prohibited since residual lytic levels may destroy the infused WU-CART-007 cells and/or prevent their in vivo expansion.

### Objectives and assessments

The primary objectives were to characterize the safety, tolerability, dose limiting toxicity (DLT), and maximum tolerated dose (MTD) or maximum administered dose (MAD; if no MTD was defined) and define the RP2D of WU-CART-007 in T-ALL/LBL (Phase 1); and to investigate the composite complete remission(CRc) rate of WU-CART-007 in R/R T-ALL/LBL patients. CRc is defined as proportion of patients that achieve a complete remission (CR) + CR with partial hematologic recovery (CRh) + CR with incomplete hematologic recovery (CRi). Secondary objectives were to assess objective response rate (ORR), defined as proportion of patients that achieve CR, CRh, CRi, morphologic leukemia free state (MLFS), and PR in patients with EMD only, and to investigate the duration of response (DOR). Exploratory objectives were to evaluate persistence and immunophenotype (cellular kinetics) of WU-CART-007 in blood and bone marrow over time; to evaluate the immunogenicity of WU-CART-007 i.e., development of anti-CAR antibodies; to understand the assessment of CD7 expression as it relates to clinical response as measured by flow cytometry; to explore the tumor microenvironment (TME) by measuring mRNA and protein levels of immune markers before and after treatment with WU-CART-007; to evaluate cytokines/chemokines levels before, during and after treatment with WU-CART-007; and to evaluate utility of minimal residual disease (MRD) in subjects achieving a CR.

Safety assessments included DLTs (in dose escalation Phase 1), adverse events, laboratory results, vital signs, and electrocardiogram findings. Adverse events of special interest (AESI) included infusion related reactions, cytokine release syndrome, immune effector cell-associated neurotoxicity syndrome (ICANS), GvHD, and prolonged pancytopenia.

Response assessments took place on day 28 of months 1, 3, 6, 12, and 24, and were defined per modified ALL Response Assessment (19) and/or Recommendations for Initial Evaluation, Staging, and Response Assessment of Hodgkin and NHL: The Lugano Classification (20). Evaluations included bone marrow biopsy and aspirate; multiparametric flow cytometry-based MRD testing performed on bone marrow aspirates; PET or CT imaging.

### Statistical Analysis

The primary analysis was conducted once the last patient completed the initial day 28 response assessment and follow-up of ≥ 3 months. Safety as assessed by the incidence of adverse events was summarized across all dose levels. Cytokine release syndrome and neurotoxicity were graded according to the American Society for Transplantation and Cellular Therapy Consensus Grading for Cytokine Release Syndrome and Neurologic Toxicity Associated with Immune Effector Cells Criteria (21). Hemophagocytic lymphohistiocytosis/macrophage activation syndrome was graded according to the American Society for Transplantation and Cellular Therapy immune effector cell-associated HLH-like syndrome (IEC-HS) grading schema (22). All other toxicities were graded utilizing the National Cancer Institute Common Terminology Criteria for Adverse Events Version 5.0 (23).

Phase 2 implemented a Simon’s optimal 2-stage design to enroll up to 20 patients at the RP2D. The null hypothesis that the true ORR is 10% was tested against the one-sided alternative hypothesis of an ORR of 40%. This design yields a type I error rate of 5% and power of 90%.

Descriptive statistics, including mean, median, standard deviations and ranges for all continuous measures were tabulated and reported. Percentages and frequencies for all categorical measures were presented. Time to events endpoints were reported using Kaplan-Meier estimates, with 95% confidence intervals for median time to event. For ORR, the estimates and the associated 95% CI (based on the Clopper-Pearson method) in each treatment group were calculated.

Duration of response was calculated from time of initial documentation of response to time of disease relapse, progression, or death due to any cause, whichever occurred first.

### Flow Cytometry

Cryopreserved peripheral blood or bone marrow mononuclear cells were thawed, washed in phosphate-buffered saline (PBS), and stained 15 minutes at room temperature with a LIVE/DEAD Fixable Blue Dead Cell Stain kit (Invitrogen, Carlsbad, CA). Cells were then washed in PBS supplemented with 0.5% bovine serum albumin and 2 mM EDTA and incubated for 10 min at room temperature with human Fc Block and Brilliant Stain Buffer (BD Biosciences; San Jose, CA). Pre-titrated saturating dilutions of fluorochrome-labeled antibodies (Extended Data Table 6) were added, and cells incubated for 30 minutes at room temperature. Intracellular staining was performed using a transcription factor staining buffer set (Thermo Fisher Scientific, Waltham, MA) according to the manufacturer’s instructions. Fluorescence minus one controls were used to assess background fluorescence intensity and set gates for negative populations. Differences in human lymphocyte antigen (HLA) class I expression between WU-CART-007 infusion products and patients, as well as WU-CART-007 expression of human CD34, were used to distinguish WU-CART-07 from the patients endogenous T cells. Samples were analyzed on a ZE5 flow cytometer (Bio-Rad, Hercules, CA). Single stain compensation controls were obtained using UltraComp eBeads (Thermo Fisher Scientific) and data were analyzed using FCS Express (DeNovo Software, Pasadena, CA). Absolute cell numbers were calculated by multiplying the adjusted white blood cell count (white blood cell count minus the absolute neutrophil count to account for Ficoll-Paque purification prior to banking) by the percentage of each subset within the CD45^+^ cell population.

### Minimal Residual Disease (MRD) Assay

Heparinized bone marrow aspirate specimens from screening, day 28 and month 3 were analyzed by flow cytometry for persistence of immunophenotypically abnormal immature T cell populations at Screening and after treatment at day 28 and month 3 for evidence of residual leukemia (24). Disease phenotype determined flow cytometry assay using BD FACSLyric using anti-human monoclonal antibodies to leukemic markers (cCD3, sCD3, CD4,CD5, CD7, CD8, CD16, CD34, CD28, CD45, CD48, CD56, CD71 and STYO16). Samples acquired using FACSLyric, FACSUITE software. Analysis performed using Woodlist Software. The presence of abnormal T progenitor population is determined at a lower level of detection at 0.01% and monitored in subsequent samples.

### Anti-Drug Antibody Assay

Patient sera (diluted 1:10) from pre-dose, 28 days after WU-CART-007 infusion, and later time points, up to 6 months, after WU-CART-007 infusion were incubated with WU-CART-007 cells or CAR negative WU-CART-007 cells at 4°C for 30 minutes. Cells were then washed with PBS containing 3% BSA before incubation with a saturating concentration of PE-conjugated goat IgG F(ab′)2 specific for human IgG + IgM (Jackson Immunoresearch, West Grove, PA) for 30 minutes at 4°C in the dark. Samples were analyzed on an Attune NxT flow cytometer (Thermo Fisher Scientific). Single stain compensation controls were obtained using UltraComp eBeads (Thermo Fisher Scientific) and data were analyzed using FlowJo (FlowJo, Ashland, OR).

### Anti-HLA Antibody Assay

Patient serum samples collected at screening, day 21, day 28, and month 3 were analyzed at local participating site laboratories for flow cytometry based single antigen anti-human leukocyte antigen antibody testing. Positive results were reported for specificity for Class I and Class II antigens.

## Figures and Tables

**Figure 3:**
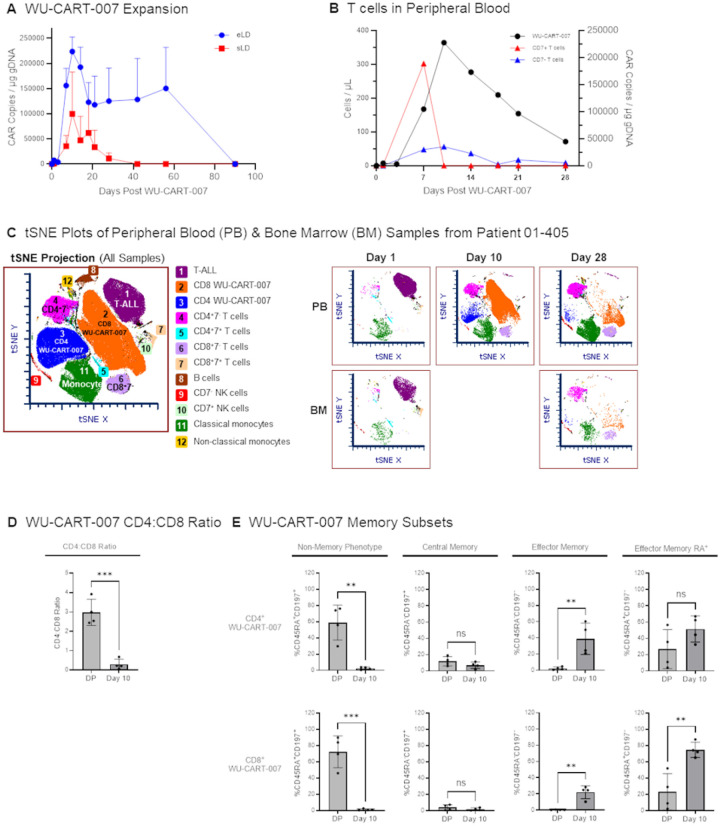
WU-CART-007 and Impact on CD7^+^ T cells. **(A)** WU-CART-007 expansion in dose level 4 patients treated with standard lymphodepletion (sLD, n = 3) and enhanced lymphodepletion (eLD, n = 12) was assessed by droplet digital PCR quantification of CAR copies in peripheral blood Lines represent mean. Error bars represent standard error of the mean. **(B)** T cell counts in peripheral blood in peripheral blood were assessed by FACS for 28 days after WU-CART-007 infusion in responsive patients treated with enhanced lymphodepletion regiment e (n = 10). Lines represent median. **(C)** tSNE projection of merged FACS files shows twelve major cell subsets (*left panel*). Peripheral blood (n = 7) and bone marrow (n = 3) samples from patient 01–405. as well as healthy PBMC controls and the WU-CART-007 infusion product, were analyzed by FACS. A merged file of all 12 samples was used to generate the tSNE projection Individual tSNE plots obtained from peripheral blood (PB) and bone marrow (BM) samples collected from patient 01–405 (*right panef*). **(D)** Expression of CD4 and COS on WU-CART-007 cells pre-infusion (DP = Drug Product) and at peak WU-CART-007 expansion (Day 10) in peripheral blood. **(E)** CD4^+^ and CD8^+^ WU-CART-007 memory subsets were assessed by FACS pre-infusion and at peak WU-CART-007 expansion in peripheral blood. Data is shown for all 900M dose patients with samples available for analysis. Bars represent mean, error bars represent standard deviation, dots represent individual patient samples (n = 4). p values <0.05 considered significant, **p < 0.01, ***p < 0.001

**Table 1:** Baseline Demographics and Disease Characteristics

Characteristic	Total (N = 26)
Age, years, median (range)	30 (14–69)
Adolescents < 18 years, n (range)	5 (14–17)
Male sex, n (%)	19 (73%)
Race, n (%)	
White	24 (92%)
Black or African American	1 (4%)
Mixed race Asian and Black or African American	1 (4%)
ECOG performance status, n (%)*	
0	10 (38%)
1	16 (62%)
2	0
Diagnosis, n (%)	
T-ALL	18 (69%)
T-LBL	8 (31%)
Prior HSCT, n (%)	10 (38.5%)
Prior nelarabine, n (%)	19 (73%)
No. of prior therapies, median (range)	4 (2–7)
Bone marrow blast count, median % (range)	50% (5–95)
Extramedullary disease only, n (%)	7 (26.9%)

ECOG, Eastern Cooperative Oncology Group; T-ALL, T-cell acute lymphoblastic leukemia; LBL, lymphoblastic lymphoma

* Medical College of Wisconsin conversion table was used to convert Karnofsky and Lansky Performance Status to ECOG

**Table 2:** Best Overall Response by WU-CART-007 Dose, Response Evaluable Patients

Response, n (%)	100M(N = 3)	300M (N = 3)	600M (N = 3)	900M sLD (N = 3)	900M eLD (N = 11)	Total (N = 23)
CRc (CR + CRi)	0	1 (33.3%)	1 (33.3%)	2 (66.7%)	8 (72.7%)	12 (52.2%)
CR	0	1 (33.3%)	1 (33.3%)	1 (33.3%)	2 (18.2%)	5 (21.7%)
CRi	0	0		1 (33.3%)	6 (54.5%)	7 (30.4.0%)
PR	0	1 (33.3%)	0	0	2 (18.2%)	3 (13.0%)
ORR (CR + CRi + PR)	0	2 (66.7%)	1 (33.3%)	2 (66.7%)	10 (90.9%)	15 (65.2%)
DOR, month, median (95% CI)	-	-	-	-	Not Reached (0.5, NE)	6.6 (1.8, NE)
Duration of follow-up, month, median (95% CI)	-	-	-	-	Not Reached (1.4, NE)	11.5 (2.7, NE)
Bridged to HCT, n	0	0	1	1	5	7

M, million; sLD, standard lymphodepletion; eLD, enhanced lymphodepletion; CRc, composite complete remission rate; CR, complete remission; CRi, complete remission with incomplete hematologic recovery; PR, partial response (extramedullary disease only); ORR, objective response rate; DOR, duration of response; CI, confidence interval; HCT, hematopoietic cell transplant; NE, not estimable; EFS, event free survival; OS, overall survival

**Table 3:** Related Treatment-emergent Adverse Events (All Eligible Patients Treated, N = 26)

	n (%)	n (%)
	All grades	Grade ≥ 3
Total number of patients with TRAEs	25 (96.2)	16 (61.5)
**Treatment-related AEs (> 10% incidence)**		
**Immune system disorders**		
Cytokine release syndrome	23 (88.5)	5 (19.2)
Hemophagocytic lymphohistiocytosis	4 (15.4)	3 (11.5)
**Investigations**		
International normalized ratio increased	9 (34.6)	0
Blood fibrinogen decreased	8 (30.8)	3 (11.5)
Blood bilirubin increased	6 (23.1)	3 (11.5)
Lymphocyte count decreased	5 (19.2)	5 (19.2)
White blood cell count decreased	5 (19.2)	5 (19.2)
Neutrophil count decreased	4 (15.4)	4 (15.4)
Platelet count decreased	3 (11.5)	3 (11.5)
Activated partial thromboplastin time prolonged	4 (15.4)	0
Alanine aminotransferase increased	4 (15.4)	2 (7.7)
Aspartate aminotransferase increased	4 (15.4)	1 (3.8)
Gamma-glutamyl Transferase increased	3 (11.5)	3 (11.5)
**Metabolism and nutrition disorders**		
Decreased appetite	4 (15.4)	4 (15.4)
Hypocalcemia	3 (11.5)	0
Hypokalemia	3 (11.5)	1 (3.8)
Hyponatremia	3 (11.5)	2 (7.7)
Hypophosphatemia	3 (11.5)	0
**Blood and lymphatic system disorders**		
Anemia	7 (26.9)	6 (23.1)
Febrile neutropenia	5 (19.2)	5 (19.2)
**General disorders and administration site conditions**		
Pyrexia	6 (23.1)	0
Fatigue	4 (15.4)	1 (3.8)
Chills	3 (11.5)	0
**Gastrointestinal disorders**		
Diarrhea	4 (15.4)	0
Nausea	3 (11.5)	1 (3.8)
**Vascular disorders**		
Hypotension	3 (11.5)	1 (3.8)
**Nervous system disorders**		
Headache	5 (19.2)	1 (3.8)

AE, adverse event; TRAE, treatment related adverse event

**Extended Data** Table 1. **WU-CART-007 Dose Levels**

## Data Availability

Upon request and subject to review, Wugen will provide the data that support the findings of the present study.
